# Coordinating Development: How Do Animals Integrate Plastic and Robust Developmental Processes?

**DOI:** 10.3389/fcell.2019.00008

**Published:** 2019-02-06

**Authors:** Christen K. Mirth, Alexander W. Shingleton

**Affiliations:** ^1^School of Biological Sciences, Monash University, Clayton, VIC, Australia; ^2^Department of Biological Sciences, University of Illinois at Chicago, Chicago, IL, United States

**Keywords:** morphogenetic growth, environmentally-sensitive growth, patterning, phenotypic plasticity, nutrition, eco-evo-devo, robustness

## Abstract

Our developmental environment significantly affects myriad aspects of our biology, including key life history traits, morphology, physiology, and our susceptibility to disease. This environmentally-induced variation in phenotype is known as plasticity. In many cases, plasticity results from alterations in the rate of synthesis of important developmental hormones. However, while developmental processes like organ growth are sensitive to environmental conditions, others like patterning – the process that generates distinct cell identities – remain robust to perturbation. This is particularly surprising given that the same hormones that regulate organ growth also regulate organ patterning. In this review, we revisit the current approaches that address how organs coordinate their growth and pattern, and outline our hypotheses for understanding how organs achieve correct pattern across a range of sizes.

## Introduction

Animals vary in their adult body size according to the environmental conditions in which they develop (Angilletta et al., 2004; Hietakangas and Cohen, 2009; Harrison and Haddad, 2011; Nijhout et al., 2014). For example, reducing the quantity or quality of food during development typically reduces body size in most animals, including humans (Stinson, 1992). This change in size results from modifications of the systemic signals that coordinate the growth of organs and the whole body (Hietakangas and Cohen, 2009). Remarkably, regardless of their final size, organs retain the same overall pattern: adult humans have the same number of teeth regardless of how large or small their head is. This has long been interpreted as meaning that the developmental patterning programs that establish cell identity are scale-free, thus independent of those that regulate organ growth (Umulis and Othmer, 2013). However, recent studies suggest that the processes of growth and patterning are far more intertwined than previously thought, and that robustness of final organ pattern is underpinned by surprising plasticity in the rate of pattern formation. Here, we review the literature that has led to this notion of plastic growth and robust pattern, and present recent evidence that this dichotomy might not accurately represent how development progresses. Finally, we outline how these insights form a generalizable framework for understanding how animals coordinate patterning processes with growth such that final pattern is robust regardless of organ size.

## Growth Responds to Developmental and Environmental Conditions

**FIGURE 1:**
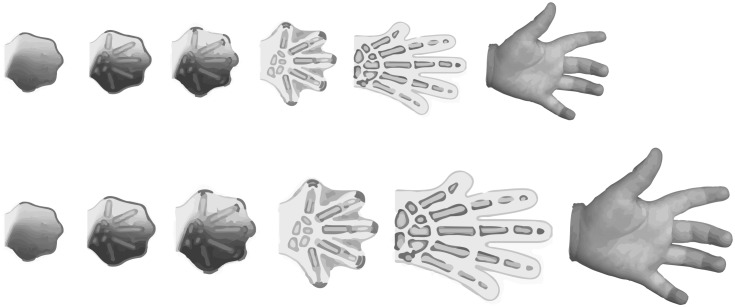
While growth of the hand may differ between individuals, due to genetic or environmental factors, the number of digits on a normally-developing hand do not. This illustrates that the hand formation requires the integration of both plastic and robust developmental processes.

Cells do not grow and divide in isolation. Rather, cell growth and proliferation is conditional and depends on neighboring cells, diffusing morphogens, circulating hormones, and the surrounding biotic and abiotic environment. Conceptually, the conditions that regulate cell growth and proliferation can be divided into “developmental” and “environmental.” Growth regulation has been studied extensively in insects that undergo complete metamorphosis, the holometabolous insects, and these different types of growth are often referred to as intrinsic morphogenetic growth and extrinsic environmentally-sensitive growth, respectively (Truman et al., 2006). Broadly speaking, morphogenetic growth can be thought of as the growth that ensures organs and tissues are the correct shape for their function, while environmentally-sensitive growth ensures organs and tissues are the correct size for their environment. For example, morphogenetic growth generates hands with five fingers, while environmentally-sensitive growth generates hands that are the right size for the environment in which the hand (and the body it is attached to) develops (Figure 1). Thus, morphogenetic growth may be considered to be robust, while environmentally-sensitive growth is plastic.

While growth has not formally been categorized as morphogenetic versus environmentally-sensitive in animals other than insects, researchers have tended to implicitly focus on one or the other. In particular, there is an extensive literature spanning decades describing how patterns of cell proliferation and growth during development generates functional organs, as is evident by review of any undergraduate developmental biology textbook. Slightly less extensive, but equally important, is the literature describing the developmental-genetic mechanisms through which environmental factors, particularly nutrition, influence body and trait size (see Gokhale and Shingleton, 2015; for review). Although much of this literature is focused on insects, which have proven highly tractable models for understanding growth and patterning, the concepts of morphogenetic and environmentally-sensitive growth may be applied to animals in general.

Morphogenetic and environmentally-sensitive growth can therefore be distinguished phenomenologically as generating different aspects of a morphology (shape versus size), and distinguished in terms of research foci within the field of developmental biology. Whether they represent two distinct developmental processes is, however, an open question, and one that we will begin to address here. Nevertheless, as a “null hypothesis” for future research, it is useful to begin by considering morphogenetic and environmentally-sensitive growth as mechanistically different processes.

### Morphogenetic Growth

In holometabolous insects, most adult organs develop during the larval period from groups of epidermal cells called imaginal discs. Almost all of the imaginal discs in Diptera (Cyclorrhapha), and the wing discs in Lepidoptera and Coleoptera, are specified early in development, and undergo both growth and patterning throughout the larval instars. For decades, these imaginal discs have proven to be convenient tools for understanding the processes that regulate cell cycle, the acquisition of cell identities, and the hormonal mechanisms controlling growth and differentiation.

**FIGURE 2:**
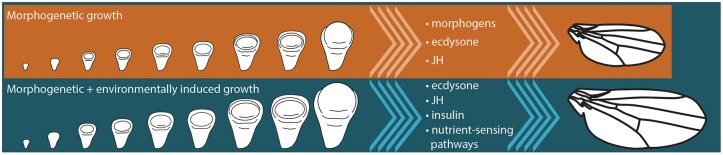
Final size of insect organs, like the wing, is a product of morphogenetic and environmentally-sensitive growth. Signaling pathways that are thought to regulate morphogenetic growth in insects include morphogen-induced signaling, as well as the signaling pathways responding to the systemic hormones, juvenile hormone (JH) and ecdysone. Environmentally-sensitive growth is regulated by a number of systemic signals, including JH, ecdysone, and insulin signaling, as well as cell-autonomous nutrient-sensing pathways like the Target of Rapamycin pathway.

The term morphogenetic growth was first applied to the growing wing disc in the tobacco hornworm, *Manduca sexta* (Truman et al., 2006). Starving *Manduca* caterpillars in the final instar inhibits growth of the body and of the wing imaginal discs (Truman et al., 2006). However, if the gland that produced an important developmental hormone, juvenile hormone (JH), is removed, the wing discs continue to grow even if larvae are starved (Truman et al., 2006). These experiments revealed two important characteristics of disc growth: (1) there is a component of growth that does not depend on nutrition, and (2) under starvation conditions JH represses this nutrition-insensitive growth. Because this nutrition-insensitive growth is thought to be driven largely by the developmental processes that generate the shape and cell identities of individual traits, it was called morphogenetic growth (Figure 2).

**FIGURE 3:**
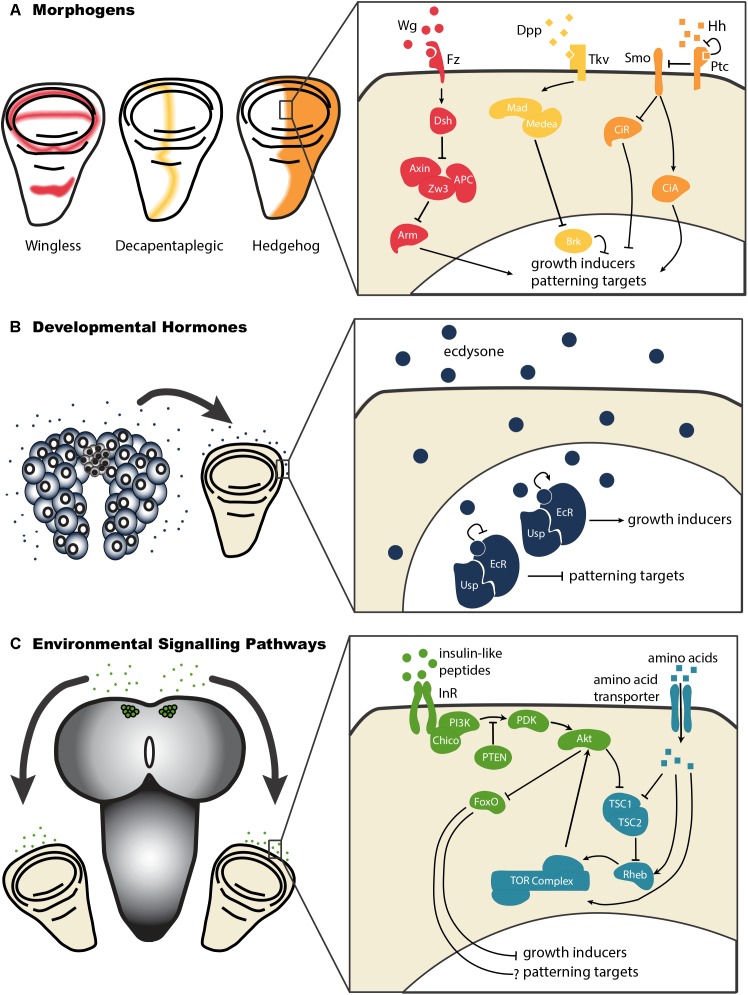
Morphogens, developmental hormones, and environmentally-sensitive signaling pathways each exert effects on growth and patterning of the wing disc. **(A)** The morphogens Decapentaplegic (Dpp), Hedgehog (Hh), and Wingless (Wg) act through morphogen-specific signaling pathways to regulate the growth and patterning of the developing wing. Abbreviations: Frizzled (Fz), Dachshund (Dsh), Zeste White 3 (Zw3), Adenomatous Polyposis Coli (APC), Armadillo (Arm), Thick Veins (Tkv), Mother’s Against Dpp (Mad), Brinker (Brk), Smoothened (Smo), Patched (Ptc), Cubitus Interruptus – Activator (CiA), Cubitus Interruptus – Repressor (CiR). **(B)** Developmental hormones like ecdysone act to regulate growth and patterning in the wing disc. By binding to its receptor, a heterodimeric complex of Ecdysone Receptor (EcR) and Ultraspiracle (Usp), ecdysone stimulates growth of the wing disc. It further relieves the repression of patterning imposed by unliganded EcR/Usp complexes in early third instar wing discs. **(C)** Environmental conditions affect wing disc growth by acting systemically, via the insulin-like peptides, or on cell autonomous nutrient sensing pathways like the Target of Rapamycin (TOR pathway). To date, we know little about how insulin and TOR signaling affect patterning in the wing disc. Abbreviations: Insulin Receptor (InR), Phosphatidylinositide 3-Kinase (PI3K), 3 Phosphoinositide-Dependent protein Kinase (PDK), Phosphatase and Tensin homolog (PTEN), Forkhead Box O (FoxO), Tuberous Sclerosis Complex 1/2 (TSC1/2), Ras Homolog Enhanced in Brain (Rheb).

While JH represses morphogenetic growth in *Manduca*, a second developmental hormone plays a role in stimulating growth in several insects. The steroid hormone ecdysone is mostly known for inducing the molts between larval instars and for driving the morphogenetic changes during metamorphosis that generate a fully-formed adult. However, it also plays an important role in growth of the imaginal discs in *Manduca*, the buckeyed butterfly *Junonia coenia*, and the fruit fly *Drosophila melanogaster* (Figure 3). Increasing the concentration of ecdysone in *ex vivo* cultures of *Manduca* or *Junonia* wing discs increases their proliferation (Nijhout and Grunert, 2002, 2010; Nijhout et al., 2007, 2018). Genetically reducing ecdysone signaling in *Drosophila*, either by manipulating or ablating the gland that produces ecdysone, results in reduced wing disc growth (Parker, 2011; Herboso et al., 2015; Moeller et al., 2017). Feeding ecdysone to *Drosophila* larvae with reduced ecdysone synthesis restores growth of the wing in a dose-dependent manner (Parker, 2011; Herboso et al., 2015).

How these developmental hormones regulate morphogenetic growth is not entirely clear, although evidence suggests that they may achieve this via the regulation of morphogens (Brennan et al., 1998; Cranna and Quinn, 2009; Mitchell et al., 2013; Djabrayan and Casanova, 2016), which drive organ-autonomous disc growth (Figure 2). Morphogens are molecules that diffuse from source cells into surrounding tissues and establish concentration gradients. In doing so, these morphogens regulate the growth and patterning of the imaginal discs. The *Drosophila* wing imaginal disc produces several morphogens, including Wingless (Wg), Hedgehog (Hh), and Decapentaplegic (Dpp), which act to regulate disc growth and patterns of cell identity. Each of these morphogens originates from a specific subset of wing disc cells, and diffuses across the wing disc (Figure 3). The gradients morphogens produce allow the specification of distinct cellular types at particular distances from the source, with specific transcription factors being activated (or de-activated) in a concentration-dependent manner. Morphogens thus specify cell fate across a field of undifferentiated cells.

How morphogens work to establish robust cell fates across genetic backgrounds and environmental conditions has been explored in depth in the nematode *Caenorhabditis elegans.* The vulva of *C. elegans* is specified in response to the activity of the LIN-3/Epidermal Growth Factor (EGF) morphogen (Sternberg and Horvitz, 1986; Katz et al., 1995, 1996). Here, the uterine anchor cell secretes the LIN-3/EGF ligand, which acts on three of six competent P cells to induce vulval cell fate. While all seven P cells are capable of differentiating into vulval cells, the central P cells - termed P5.p, P6.p, and P7.p – adopt vulval fate while the remaining P cells adopt an alternate fate (Sulston and White, 1980; Kimble, 1981; Sternberg and Horvitz, 1986). This is because the P6.p cell is closest to the anchor cell, and thus receives the highest dose of LIN-3/EGF. High levels of EGF receptor activation cause the P6.p cell to adopt the primary vulval cell fate. The P6.p cell in turn inhibits the primary cell fate in its P5.p and P7.p neighbors via the LIN-12/Notch pathway (Newman et al., 1995; Levitan and Greenwald, 1998). These cellular interactions establish a stereotyped pattern of cell divisions that will lead to the formation of a functional vulva.

Vulval cell specification is remarkably consistent across environmental conditions. When rearing nematodes across a range of thermal, nutritional, and media conditions, Braendle and Félix found that 97.65% of individuals had the correct cell fate pattern (Braendle and Félix, 2008). Only 0.25% of individuals demonstrated non-functional vulval patterns, while 2.1% showed non-canonical yet complete vulval patterns. Further, while environmental conditions significantly altered gene expression patterns in the vulval fate specification pathway, this rarely resulted in phenotypic change (Braendle and Félix, 2008). This is because correct vulval cell fate patterns are induced across a wide range of LIN-3/EGF pathway signaling activities (Barkoulas et al., 2013). If we extrapolate these findings to other animals, we could hypothesize that morphogens confer robustness by reliably inducing cell fates across the range of environmental conditions to which organ growth shows sensitivity.

In principle, this could be achieved if morphogens specified cell fate after cell proliferation has finished. The most obvious aspect of patterning in the adult wing in insects is the position of the veins, and this is regulated by Dpp (as well as other genes) in the wing imaginal disc. Uniform expression of Dpp across the wing disc eliminates vein formation (Bosch et al., 2017). However, complete loss of Dpp inhibits imaginal disc growth (Bosch et al., 2017). Thus, Dpp is necessary for disc growth while the Dpp gradient is necessary for disc patterning. The differences in the mechanisms through which Dpp gradients induce patterning while growth remains gradient-independent may be key to understanding how these processes are coordinated.

Regardless of how Dpp achieves these two functions, its dual role as a regulator of both patterning and growth ensures that these processes occur at the same time. This phenomenon is also observed during growth and patterning of the mammalian limb (Figure 1). Here, the limb generates an increasing number of lateral bones as it extends from the proximal stylopod (e.g., femur) to the distal autopods (e.g., digits). This patterning involves the spread of two morphogens, Bone Morphogenetic Protein2 and Wnt, which – together with the transcription factor sox9 – form a Turing network to specify the position of pre-cartilage (Raspopovic et al., 2014). At the same time Wnt works with a third morphogen, Fibroblast Growth Factor, to stimulate cell proliferation and limb elongation (Yamaguchi et al., 1999; Gros et al., 2010). The development of both the wings of insects and limbs of mammals illustrate how the processes of growth and pattern are intimately linked, and the central role of morphogens in maintaining this link.

### Environmentally-Sensitive Growth

Environmentally-sensitive growth generates traits of different sizes under different environmental conditions. Many environmental factors affect growth, including nutrition, temperature, and oxygen level (Mirth and Shingleton, 2012). Whether these factors each have distinct mechanisms through which they regulate growth, or whether there are signaling pathways common to all environmentally-sensitive growth remains unclear.

There are two conceptually different ways that an environmental factor can influence growth. The first is through systemic mechanisms, where the developing animal senses the environment and responds by regulating growth through the endocrine or nervous system or both. The second is through cell-autonomous mechanisms, where the environment impinges directly on dividing cells to regulate their rate of growth and proliferation.

Perhaps the best elucidated example of the systemic control of environmentally-sensitive growth is the effect of nutrition on growth rate via the insulin/insulin-like growth factor signaling (IIS) pathway, a pathway conserved amongst all animals (Hietakangas and Cohen, 2009). In insects like *Drosophila*, high concentration of protein in the larval diet stimulates the release of insulin-like peptides into the circulation (Nijhout et al., 2014). Insulin-like peptides bind to insulin receptors in the cells of target organs. This activates the IIS pathway thereby regulating cell growth and proliferation, primarily by de-repressing growth inhibitors.

Protein concentration in the larval diet can also influence cell growth and proliferation through cell-autonomous mechanisms. For example, the Target of Rapamycin (TOR) signaling pathway responds directly to cellular levels of amino acids (Saxton and Sabatini, 2017), which in turn reflect levels of circulating amino acids, to regulate cell and organ growth. Thus, a single environmental factor may influence growth rate through multiple systemic and cell-autonomous mechanisms.

The length of time an animal spends growing is also regulated by environmentally-sensitive systemic signals, further contributing to final organ and body size. Because final organ and body size is the product of growth rate and duration, different environmental factors may have opposite effects on growth rate, but may have the same effect on final body and trait size. For example, both low nutrition and high temperature decrease adult size in the tobacco hornworm *Manduca sexta*, but the former reduces growth rate while the latter increases it (Davidowitz et al., 2004). This is because low nutrition increases growth duration less than it decreases growth rate, while high temperature decreases growth duration more than it increases growth rate (Davidowitz et al., 2004). In both cases the net effect is a reduction in final size. Thus, for animals to control their size response to environmental change, they must carefully control the duration of environmentally-sensitive growth.

Ecdysone is well-establish as the gate keeper for developmental transitions in insects, inducing the molts between instars, the onset of metamorphosis, and the development of adult tissues. At several stages of larval development, environmental conditions like nutrition can alter the timing of ecdysone synthesis itself (Mirth and Shingleton, 2012). The final larval instar of *Drosophila* has three pulses of ecdysone responsible for initiating new phases of the larval developmental program (Warren et al., 2006). The first of these pulses is known as the critical-weight ecdysone-pulse and is sensitive to environmental conditions, particularly to temperature and nutrition (Mirth et al., 2005; Shingleton et al., 2005; Ghosh et al., 2013; Koyama et al., 2014). Starving larvae dramatically delays the critical-weight ecdysone-pulse, thereby extending the length of the larval growth period (Koyama et al., 2014). Delays in ecdysone synthesis arise in response to reduced activity of the IIS and TOR pathways in the ecdysone-producing gland, the prothoracic gland (Caldwell et al., 2005; Colombani et al., 2005; Mirth et al., 2005; Layalle et al., 2008; Koyama et al., 2014).

The critical-weight ecdysone-pulse further changes the way that growth is regulated. Before this pulse, the growth of the developing wing discs and ovaries is more sensitive to nutrition than after the pulse (Shingleton et al., 2008; Mendes and Mirth, 2016). In the ovaries, this is because insulin signaling regulates ovary growth before the critical weight ecdysone pulse, while after the pulse, ecdysone and insulin signaling both regulate ovary growth rendering this organ less sensitive to nutrition (Mendes and Mirth, 2016). The growth of wing discs in *Manduca* and *Junonia* also change their sensitivity to starvation with critical weight (Miner et al., 2000; Tobler and Nijhout, 2010), although the underlying mechanism for the change in sensitivity in these species is not known. Collectively, however, these data suggest that particular pulses of ecdysone, which are themselves environmentally sensitive, contribute to the onset of morphogenetic growth in *Drosophila*.

While hormones regulate the extent to which environmental conditions influence growth rate, traits differ in their autonomous response to the same environmental growth regulator. For example, in many animals, growth of both the genitalia and the brain is relatively insensitive to changes in nutrition (Shingleton et al., 2005; Cheng et al., 2011; Tang et al., 2011). The result is that as overall body size declines with decreasing nutrition, the genitals and brain are “spared,” and are more or less the same size regardless of nutritional conditions. In *Drosophila*, this is because both tissues are able to maintain IIS activity when levels of circulating insulin-like peptides are low, and so are relatively insulin-insensitive. The opposite pattern is seen in secondary sexual characteristics used by males to compete for or attract mates, which are proportionally larger in larger individuals. Such traits are more sensitive to changes in nutrition than other body parts, and in rhinoceros beetle at least, this is due their heightened insulin-sensitivity (Emlen et al., 2012).

Similar differences in environmental sensitivity are seen among *Drosophila* traits in response to changes in temperature, such that temperature has less of an effect on the rate of cell proliferation in the wing than in the leg (McDonald et al., 2018). Counterintuitively, because of the opposing effects of temperature on growth rate and growth duration, this reduction in the thermal plasticity of growth rate in the wing makes final wing size more thermally plastic than other traits, and results in the wings being proportionally larger at lower temperatures (McDonald et al., 2018). This decreases wing loading and increases flight efficiency when wing beat frequency is slowed at cooler temperatures (Frazier et al., 2008).

## Integrating Plastic and Robust Developmental Processes

Ostensibly, therefore, there are two classes of developmental mechanisms that regulate growth: mechanisms that generate morphogenetic growth through patterning and mechanisms that generate environmentally-sensitive growth by responding either systemically or cell-autonomously to environmental signals. How then are these two classes of mechanisms integrated?

The simplest model is that environmentally-sensitive signaling pathways, such as IIS and TOR-signaling, regulate the morphogenetic growth and organ patterning pathways, such as Dpp- and Hh-signaling. For example, if the spread of Dpp in the wing imaginal disc of *Drosophila* were regulated by IIS, either by IIS regulating the production of Dpp or by regulating the diffusion of Dpp across the disc, this would generate correctly patterned discs across a range of nutritional environments. While the effect of IIS on Dpp signaling has not yet been explored, there is evidence that the DPP gradient scales with disc size – that is the relative gradient is unaffected by disc size – when disc size varies with age (Wartlick et al., 2011) and due to disc-autonomous changes in IIS (Teleman and Cohen, 2000). However, IIS is not the only environmental regulator of wing disc size in *Drosophila*, and it seems unlikely that each environmental regulator of size influences the Dpp gradient independently. Rather, the gradient may reflect wing disc size itself, regardless of the environmental factors that regulate disc growth (Ben-Zvi et al., 2011; Parker, 2011; Umulis and Othmer, 2013). That is, patterning and morphogenetic growth accommodate environmental changes in growth rate.

An alternative model is that environmental and morphogenetic regulators of size are integrated at the level of cell proliferation. Recently, Parker and Struhl published data supporting this hypothesis (Parker and Struhl, 2015). They found that TOR signaling is a regulator of Warts/Hippo-regulated growth. The Warts/Hippo signaling regulates cell proliferation by inhibiting the activity of the growth-promotor Yorkie (Parker and Struhl, 2015). Importantly, the activity of the Warts/Hippo-signaling pathway both regulates and is regulated by various morphogens, including Dpp (Rogulja et al., 2008) and Wg (Zecca and Struhl, 2010). They, and other authors, therefore propose that Warts/Hippo signaling is the integrator of morphogenetic and environmentally-sensitive growth (Gotoh et al., 2015).

**FIGURE 4:**
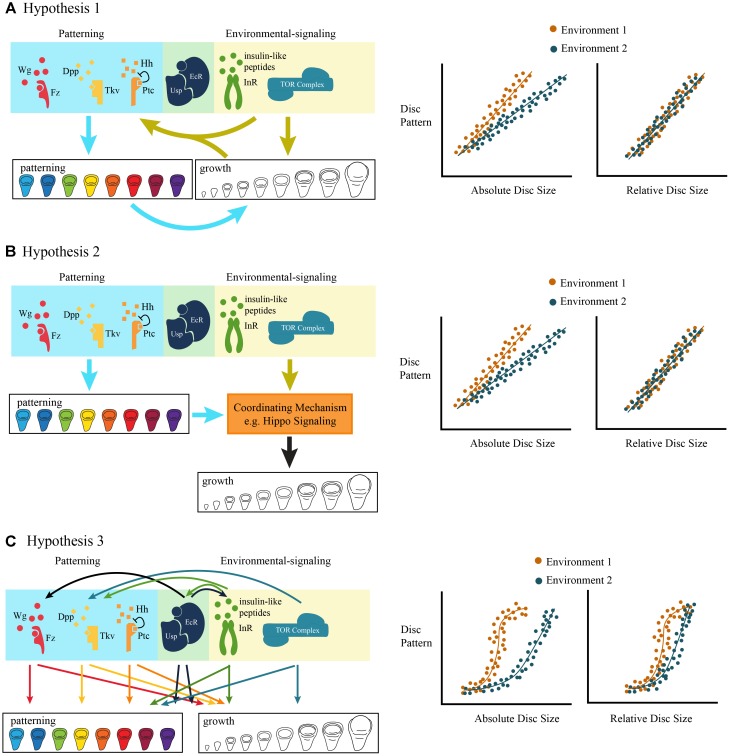
Three non-mutually exclusive models of the coordination of morphogenetic and environmentally-sensitive growth. **(A)** Hypothesis 1: Environmental signals regulate growth both via environmentally-sensitive signaling pathways, and via patterning signaling pathways. The effect of the environment on patterning is either direct, via environmentally-sensitive signaling pathways, or indirect, via the environmentally-regulated size of the imaginal disc. The ontogenetic relationship between disc size (relative to final size) and pattern is stereotyped and maintained across environmental conditions. **(B)** Hypothesis 2: Both environmental and patterning signals are coordinated by a single mechanism, possibly Hippo signaling, to drive growth. Again, the ontogenetic relationship between disc size (relative to final size) and pattern is stereotyped and maintained across environmental conditions. **(C)** Hypothesis 3: There is a complex relationship between pattern and growth, such that multiple patterning and environmentally-sensitive signaling pathways crosstalk, potentially independently from one another. The ontogenetic relationship between disc size (relative to final size) is complex and rates of pattern formation vary across environmental conditions.

If both environmental signals and morphogenetic signals regulate cell proliferation via a common downstream mechanism, then we might expect growth and patterning to be tightly coordinated. In this case, the temporal dynamics of patterning and growth should progress synchronously, resulting in a stereotypical relationship between organ size and organ patterning (Figure 4). Environmental conditions that generate variation in final trait size would cause the trajectories to be scaled to organ size, but would not change the shape of the relationship between organ size and patterning.

Testing this hypothesis requires the quantification of both the spatial and temporal changes in patterning as the organ develops and the growth of the organ over developmental time. In *Junonia*, the trachea that fill the wing veins show distinguishable stages of differentiation throughout the final larval instar that can be used to define stages of disc patterning. Here, the stages of disc patterning progress linearly with increasing disc size (Miner et al., 2000). Altering the rate of growth of the wing discs, by starving the caterpillars, changes the size at which a disc reaches a patterning stage, but does not change the trajectory of the disc size/disc pattern relationship (Miner et al., 2000). This suggest that in *Junonia* wing discs, the mechanisms integrating robust versus plastic development might occur via the regulation of a common downstream mechanism.

This synchronization between growth and patterning does not appear to be universally true. In a study of the progression of patterning in the *Drosophila* wing imaginal discs under different environmental conditions, Oliveira et al. found considerable “slippage” in the rate at which patterning progressed for different patterning genes (Oliveira et al., 2014). That is, environmental change differed in its impacts on patterning rates across gene products (Oliveira et al., 2014). In this case, patterning and environmentally-sensitive growth do not appear to be tightly coordinated. Even so, regardless of how environmental conditions affected the trajectory of patterning, all the discs attained the “correct” final pattern by the end of development (Oliveira et al., 2014).

The fact that patterning genes responded differently to the same environmental variable indicate that the progression of patterning, and presumably morphogenetic growth, is itself environmentally sensitive, independent of disc size (Figure 4). This can be explained in part because morphogens respond to different signals from the developmental environment. While TOR and Dpp interact by regulating common downstream components (Parker and Struhl, 2015), other morphogens receive input from alternative signaling pathways. In the early third instar, Wg expression across the wing margin, in the wing pouch, and in the region that will give rise to dorsal thoracic structures depends on ecdysone signaling (Mirth et al., 2009; Herboso et al., 2015). Either starvation, which delays the critical weight ecdysone pulse, or suppression of ecdysone synthesis in early third instar larvae inhibits the expression of Wg in the wing imaginal discs (Mirth et al., 2009). Starvation or suppression of ecdysone synthesis also prevents the expression of Cut, Senseless, and Achaete (Mirth et al., 2009; Herboso et al., 2015), known Wg targets (Neumann and Cohen, 1996; Johnston and Sanders, 2003). Finally, ecdysone signaling acts via Wg to regulate patterns of cell division at the end of the third instar (Couso et al., 1994; Mitchell et al., 2013; Guo et al., 2016). Thus, while environmental conditions coordinate TOR with Dpp activity through Hippo-signaling, they regulate Wg signaling activity by modulating the timing of ecdysone pulses.

In the ovary, the coordination of environmentally-sensitive growth and patterning involves inputs from two systemic signals, ecdysone and IIS. Early in the third instar, terminal filament cells differentiate from the unspecified somatic cells, which will later organize into the stacks of cells that define the position of each ovariole – the egg-producing units of the ovary (Godt and Laski, 1995; Sahut-Barnola et al., 1995; Sahut-Barnola et al., 1996; Hodin and Riddiford, 1998; Sarikaya et al., 2012). Terminal filament cell differentiation is marked by the onset of Engrailed (Forbes et al., 1996), which is induced by the critical weight ecdysone pulse (Mendes and Mirth, 2016). Starving larvae before the critical weight ecdysone pulse, or suppressing ecdysone signaling in the ovary, delays Engrailed expression and ovary growth (Mendes and Mirth, 2016). In addition, reducing ecdysone signaling reduces the rate of terminal filament stack formation. Similarly, downregulating insulin signaling in the ovary delays the onset of Engrailed expression and reduces the rates of terminal filament stack formation (Mendes and Mirth, 2016). This illustrates how developmental processes like the onset of cell differentiation and ovary growth depend on multiple systemic signals.

Collectively, the data therefore suggest a more complex relationship between morphogenetic and environmentally-sensitive growth, with common downstream components (e.g., Hippo-signaling); cross talk between canonical patterning mechanisms (e.g., Wg-signaling) and environmentally-sensitive mechanisms (e.g., ecdysone-signaling); and potentially direct environmental regulation of morphogenetic signaling. To add to this complexity, the activity of morphogenetic and environmentally-sensitive signaling pathways changes over time. The expression domains of Wg in the *Drosophila* wing disc changes over larval development (Mirth et al., 2009; Oliveira et al., 2014), potentially affecting how this morphogen affects growth and patterning. Further, the relative roles of insulin and ecdysone signaling in regulating the growth and patterning of wing discs and ovaries also changes over developmental time (Shingleton et al., 2008; Mirth et al., 2009; Mendes and Mirth, 2016). This suggests that for some organs multiple changing inputs act to regulate growth and pattern.

This interaction between multiple inputs is likely to be key to conferring robustness of pattern. Generally, two types of signaling pathway configurations are thought to lead to robustness in developmental systems: redundant pathway configurations or distributed pathway configurations (Wagner, 2005; Félix and Wagner, 2008). Redundant pathway configurations result when multiple gene products perform the same function, such that each induces the same downstream effect in a pathway. This generates robustness since the perturbation of any one gene product will not alter the output of the pathway, so long as the remaining inputs remain above the threshold to sustain the output. For example, during *Drosophila* embryogenesis the gene duplicates *knirps* and *knirps-related* are both sufficient for inducing head development, but neither gene is necessary (Gonzalez-Gaitan et al., 1994). It is worth noting that gene duplication typically results in divergence of function in each duplicate, such that the copies are rarely ever truly redundant in function (Force et al., 1999). Although *knirps* is not necessary for head development, it is required for abdominal development (Gonzalez-Gaitan et al., 1994). This example raises the question of whether redundancy figures significantly in regulating developmental robustness (Wagner, 2005; Félix and Wagner, 2008).

In distributed pathway configurations, the components of the pathway each perform distinct functions, with each function converging on the same output (Wagner, 2005; Félix and Wagner, 2008). Pathways that show distributed configurations tend to involve extensive feedback loops between the alternate routes within the pathway. These feedback loops provide the potential for non-linear dynamics, thought to be necessary to maintain homeostatic control over the output of the pathway thus ensuring its robustness (Wagner, 2005; Félix and Wagner, 2008). Because the various pathways that regulate growth and patterning interact, presumably at multiple points, this provides the potential for non-linear dynamics.

How non-linear dynamics confer robustness has been extensively studied in metabolic cascades, which are renowned for being driven by complex cycles (Félix and Wagner, 2008; Félix and Barkoulas, 2015; Nijhout et al., 2017). An interesting effect created by the structure of these cascades is that although the output of metabolism is robust to environmental conditions, the intermediate steps in the synthesis of metabolites is highly sensitive to environmental perturbation (Félix and Barkoulas, 2015; Nijhout et al., 2017). For example, in folate one-carbon metabolism, the output – formate concentration – is highly robust against fluctuations in amino acids (Nijhout et al., 2007). However, the reactions underlying formate production vary dramatically with amino acid concentration (Nijhout et al., 2007). This illustrates that robustness is dynamically regulated, and that intermediate steps in the process show plasticity and variation in trajectory when the final phenotype does not (Nijhout and Reed, 2014; Nijhout et al., 2017).

Similar types of effects are likely to be driving robustness during development (Nijhout et al., 2017). The effects of morphogens and developmental hormones are frequently non-linear, giving rise to threshold or on/off effects for patterning genes. If the patterning and growth of an organ is regulated by multiple signaling pathways, some of which exert non-linear effects and vary in strength over developmental time, this could lead to non-linear effects on the rates of patterning and growth. In this case, we would expect to observe non-linear dynamics in the relationship between organ patterning and organ growth (Figure 4). In other words, although the final phenotype – organ patterning – may be robust against environmental perturbation, interactions between growth and patterning mechanisms results in plasticity in the trajectory of pattern relative to organ size.

## Where Do We Go From Here?

We are only beginning to devise approaches to understand how animals generate phenotypes that are robust with respect to pattern but plastic with respect to size. While it has been useful to frame the question of plasticity and robustness within the context of morphogenetic growth versus environmentally-sensitive growth, it is likely that the two processes are not distinct. Nevertheless, a good starting point is to try a define the dynamic relationship between well-understood patterning mechanisms, such as Dpp and Wg signaling, and growth, and how this relationship is affected by changes in well-understood environmental-signaling mechanisms, such as IIS/TOR-signaling. This will allow us to determine whether morphogenetic and environmentally-sensitive growth are coordinated by a few or multiple interacting mechanisms.

Secondly, few studies explore the effects of signaling pathways over developmental time, choosing instead to manipulate signaling throughout development and examine the effects on phenotype at a single time. Indeed, in contrast to our understanding of the kinetics of biochemical and physiological processes, the kinetics of developmental and patterning mechanisms are largely unknown. By determining how signaling pathways affect developmental processes as they unfold over time, we gain deeper insight into when a particular signaling pathway plays its greatest role, whether that role changes, and how signaling contributes differently to growth versus patterning. Further, an understanding of developmental and patterning kinetics is fundamental to developing mathematical models that more completely describe development (see below).

Thirdly, although studying the effects of multiple signaling pathways at once is extremely difficult, pairwise manipulations provide valuable information. Developmental biologists have used pairwise manipulation of signaling pathways for decades to determine whether genes lie in the same or parallel pathways. When combined with studies of the dynamics of patterning and growth, these types of manipulations can determine to what extent pathways overlap in function, and whether this overlap is shared between growth and patterning phenotypes.

Finally, even the simplest conceptual models of morphogenetic and environmentally-sensitive growth become complex once time is considered, inhibiting intuitive reasoning. Such complexity means that the most effective method to address the problem may be to take a systems biology approach. This would involve mathematically modeling how molecular, cellular, and hormonal networks work spatially and temporally to generate organs with robust patterning but plastic size. Such mathematical models are not solely hypotheses to be confronted with data. Well-supported models describe developmental phenomena in ways that are transparent and increase the utility of our understanding, both through practical application and through inspiring further lines of research.

## Conclusion

All animals need to coordinate patterning and morphogenetic growth with environmentally sensitive growth, to achieve correct organ function across a range of organ sizes. The mechanisms through which this occurs requires inputs from multiple levels of signaling, from organ autonomous regulators of growth and pattern, to systemic signals that control development, to environmentally-sensitive signaling pathways that match growth with the environmental conditions. Understanding how these processes work together to regulate trait size is not a very tractable problem, made more complex by the paucity of studies that explicitly address both aspects of growth regulation. Indeed, after further research, the terms morphogenetic growth and environmentally-sensitive growth may no longer prove useful, since they represent the same processes at a developmental level. While the gap in knowledge is obvious, how to fill it is not. Our suggestions for future research reflect our own biases, shaped by our particular (and limited) research programs. However, it will likely take many different approaches by researchers with diverse skill-sets to begin to more fully understand how traits can be both plastic and robust to environmental change. This review is, ultimately, a call to engage in this task.

## Author Contributions

All authors listed have made a substantial, direct and intellectual contribution to the work, and approved it for publication.

## Conflict of Interest Statement

The authors declare that the research was conducted in the absence of any commercial or financial relationships that could be construed as a potential conflict of interest.
